# Examining the teaching roles and experiences of non-physician health care providers in family medicine education: a qualitative study

**DOI:** 10.1186/s12909-015-0283-8

**Published:** 2015-02-13

**Authors:** Serena Beber, Viola Antao, Deanna Telner, Paul Krueger, Judith Peranson, Christopher Meaney, Maria Meindl, Fiona Webster

**Affiliations:** 1 Department of Family & Community Medicine (DFCM), University of Toronto (UT), 500 University Avenue, Toronto, Canada; 2 South East Toronto Family Health Team, Toronto, Canada; 3 Women’s College Hospital, Toronto, Canada; 4 St. Michael’s Hospital, Toronto, Canada; 5 7 Audley Ave., Toronto, ON M4M1P5 Canada

## Abstract

**Background:**

Primary Care reform in Canada and globally has encouraged the development of interprofessional primary care initiatives. This has led to significant involvement of non-physician Health Care Providers (NPHCPs) in the teaching of medical trainees. The objective of this study was to understand the experiences, supports and challenges facing non-physician health care providers in Family Medicine education.

**Methods:**

Four focus groups were conducted using a semi-structured interview guide with twenty one NPHCPs involved in teaching at the University of Toronto, Department of Family & Community Medicine. The focus groups were transcribed and analyzed for recurrent themes. The multi-disciplinary research team held several meetings to discuss themes.

**Results:**

NPHCPs were highly involved in Family Medicine education, formally and informally. NPHCPs felt valued as teachers, but this often did not occur until after learners understood their educator role through increased time and exposure. NPHCPs expressed a lack of advance information of learner knowledge level and expectations, and missed opportunities to give feedback or receive teaching evaluations. Adequate preparation time, teaching space and financial compensation were important to NPHCPs, yet were often lacking. There was low awareness but high interest in faculty status and professional development opportunities.

**Conclusions:**

Sharing learner goals and objectives and offering NPHCPs feedback and evaluation would help to formalize NPHCP roles and optimize their capacity for cross-professional teaching. Preparation time and dedicated space for teaching are also necessary. NPHCPs should be encouraged to pursue faculty appointments and to access ongoing Professional Development opportunities.

## Background

In order to better manage complex chronic disease in primary care, primary care health systems in Canada and globally have progressively transitioned from solo medical practices to mixed-professional, team-based approaches to care [1]. These reforms have contributed toward the development of a range of interprofessional primary care initiatives, promoting collaboration and teamwork among physicians and non-physician Health Care Providers (NPHCPs) [1-3]. They have also encouraged a critical review of the way health professional education programs prepare graduates to work effectively in interprofessional collaborative settings. Specifically, poor teamwork skills have been identified as a gap in professional education that affects health inequities and challenges health care systems in keeping up with costs and complex care demands [4]. A central recommendation forming the basis of curriculum revision is to increase and enhance the opportunities for health professionals from different disciplinary backgrounds to work and learn together [1,4,5].

Within the Canadian context, the transformation of Family Medicine teaching units to interprofessional primary care teams has led to increased opportunities for involvement of NPHCPs in cross-professional teaching of medical learners. A limited literature base on the role of NPHCPs as medical educators has described a range of benefits including enhanced residency experiences [6] and perceived value in teaching areas of clinical specialization, such as musculoskeletal assessment, or women’s health and well-baby care [7,8] . Furthermore, primary care physicians at academic teaching sites face challenges with increased patient loads and increased teaching workloads as a result of insufficient numbers of medical educators [9]. Effectively integrating NPHCPs as teachers could potentially help distribute both academic and clinical work.

The University of Toronto’s Department of Family and Community Medicine (DFCM) is the largest Family Medicine training program in North America. Each of its 14 academic teaching sites is unique in their level of urbanization, their diversity of population and unit size, however, by 2009, all units had adopted multidisciplinary teams for the delivery of primary care (see Table 1 for a list of NPHCP participants in the focus groups). All NPHCPs in this study had direct patient care responsibilities and were also involved in educating medical learners. Quantitative research by these authors previously demonstrated that 76% of NPHCPs at these academic teaching units are actively involved in primary care teaching of medical learners (medical residents and/or clinical clerks/medical students) within the DFCM [10]. Despite the variability in clinical models of care at different sites, similar challenges were experienced and identified by all NPHCPs. Specifically, a large degree of variability was reported in the level of preparation and support NPHCPs received for teaching duties. Furthermore, of the 126 NPHCPs that were surveyed who teach medical learners, 82% did not have faculty appointments within academic institutions. This limited their access to appropriate and ongoing preparation and skills training, which many voiced interest in further developing [10]. The objective of this study was to further understand qualitatively the experiences, supports and challenges facing NPHCPs in family medical education.

**Table 1:** **Health care profession representation in focus groups**

Health care profession	Number of focus group participants
**Pharmacist**	5
**Registered nurse**	3
**Social worker**	3
**Registered dietitian**	3
**Nurse practitioner**	3
**Mental health worker**	1
**Chiropodist**	1
**Physiotherapist**	1
**Speech language pathologist**	1

## Methods

This descriptive qualitative study was designed to follow a 2010 quantitative survey of 126 NPHCPs involved in teaching Family Medicine residents in the 14 DFCM teaching sites [10,11]. Recruitment for focus groups was done using purposive convenience sampling [12]. Participants in the quantitative study were asked if they would be willing to take part in a focus group to explore their teaching experience and needs in greater depth. The general approach to this study was constructivist, to gather an in-depth understanding of the perspective of our participants’ lived experiences as educators for medical trainees [13]. We recognized that the NPHCPs were from different professions and working at different Family Medicine teaching units, resulting in different experiences in teaching. This diversity was accepted and formed the lens for this study, while anticipating that common themes could be used to eventually better support NPHCPs in their roles. A semi-structured focus group interview guide was developed by the research team based on the quantitative survey findings. The guide was used to help provide direction of facilitated discussions and ensure specific information was collected (see Focus Group Semi-Structured Guide). Ethics approval was received through the University of Toronto’s Research Ethics Board, reference #25607. Informed consent was obtained by all participants.

### Focus group semi-structured guide: Topics and sample prompts

Current Teaching Roles:

1. Can you briefly describe your current teaching role? What and whom do you teach?
  Prompts: Who decides the content/format of your teaching? Has your teaching role changed over time? Why has it changed? How do you think your teaching is regarded by others?

Teaching Preparation:

1. What resources/supports do you find helpful for teaching?
2. How prepared do you feel in the following teaching formats?
  Teaching, providing feedback, clinical supervision, and small group teaching.
3. What do you think would be helpful for new teachers to support this role?

Barriers in Teaching:

1. Are there any challenges you have encountered with respect to effectively implementing your teaching roles or educational initiatives? What strategies do you use to manage these challenges?
2. Our survey found that the most common challenges to effectiveness were lack of time for teaching and lack of funding. What is your experience with these challenges? Do you concur? How could these barriers be overcome? What would be helpful?

Reflections on Survey Results and Future Plans:

1. Our survey found that 18% of NPHCP who are teaching medical learners have faculty status. What do you think about this number?
2. What are your experiences with obtaining a faculty appointment?
3. What do you think might change (if anything) if you obtained faculty status?
4. Based on the results from the survey, the project team has come up with the following recommendations or ideas about how to further support -NPHCP in their teaching roles and endeavours: increasing access to Professional Development opportunities, encouraging more NPHCP to get faculty appointments. What is your response to these recommendations?

Focus groups were conducted by two research team members (DT, MM), each with experience in focus group facilitation. Each focus group was observed by a second team member (SB, JP). The focus groups were 90 minutes in length. Focus groups were recorded, transcribed and anonymized. Data collection and analysis occurred iteratively, with discussions held after each focus group. Subtle modifications were made to the semi-structured guide based on team discussions. The first focus group transcript was coded by two independent researchers (SB, FW) and a coding framework was developed. The remaining three transcripts were coded based on this framework; NVivo software was used to manage the data. The initial codes were summarized and discussed across team meetings and a thematic analysis undertaken. The team also aimed to be reflexive by holding several meetings in which the analysis was discussed amongst the multi-disciplinary team (including physician and nonphysician teachers, a quantitative expert, a sociologist). At these meetings multiple interpretations were explored. The team determined that saturation of key themes was reached after 3 focus groups. One final focus group was conducted to confirm this assessment.

The RATS guideline for reporting qualitative research was used to ensure quality in the reporting of our study in relation to sampling, role of researchers, ethics, analysis and discussion.

## Results

Four focus groups were conducted with a total of 21 participants (Table 1). Focus groups ranged in size from 3 to 8 participants. Each group was multiprofessional. All focus group participants reported teaching learners of their own professions in addition to medical learners, however the discussions concentrated on the NPHCPs’ work with medical learners. Teaching occurred in formal (presentations/workshops) and informal (shadowing/ad hoc questioning) situations. Analysis of the data revealed three overarching themes specific to their experience as medical educators: Lack of Integration, Feeling Valued as an Educator and Competing Demands. Several sub-themes were found within the larger themes and many of the findings overlapped multiple themes. Table 2 includes representative quotes for each of the themes.

**Table 2:** **Focus group participants' quotes related to themes**

Themes	Participants’ quotes
Lack of integration	“When I was asked to teach them I really wasn’t given any information on if there was anything in particular that I was to teach them, or outlines of what they needed and that would have been helpful for me to know” (FG 4, dietitian)
	“I know very little about the curriculum … sometimes residents ask about the same things over and over again and I wonder, why is that, and then you realize it’s a key piece of their curriculum or a new entry to practice criteria, so, I do find it informative in that perspective.” (FG1, pharmacist)
	“The days that I had the residents shadow me, sometimes I wouldn’t know until a couple of days before and that’s very difficult to try to get the most appropriate patients in” (FG 3, nurse)
Feeling valued as an educator	“I’ve had to earn my stripes…. once they know that they can rely on what I’m saying…then they start throwing more stuff at me.” (FG3, nurse)
	“When you’re looking at teaching needs, it depends on what the role is. If we’re collaborators, then the biggest challenge is, I have a learner here, but they have to learn how to collaborate with me and I can’t spoon feed them too much at the wrong time” (FG1, pharmacist).
	“I think our residents love it when the [NPHCP] teach them. … When we start to team teach … they see how we interact together and it’s role modeling for them” (FG 4, pharmacist)
	[Faculty Status would provide] “acknowledgement of our role and our status… it’s an important little piece of … giving back to us” (FG3, speech language pathologist)
	“I work very hard …[there’s] no acknowledgement for it …. I don’t get anything for the hard work that I put into the medical students” (FG3, chiropodist)
	“You’re doing two jobs at the same time and teaching always takes time. You’re not being compensated for it” (FG1, Mental Health Worker)
Competing demands	“… if I can’t even give back to my own profession, how can I … educate other health care professionals …?” (FG 3, physiotherapist)
	“For anybody to recognize the value of our services, we have to be visible and if we’re not visible then they forget about us” (FG2, pharmacist
	“… honestly … the washroom [is] the only place where you can go (to debrief)” (FG 1, nurse).
	“getting protected time to work on anything, even if it’s updating our skills in something that we teach… happens after hours” (FG4, nurse)

### I. Lack of integration

#### Teaching role and format

When NPHCPs were asked to lead formal presentations and workshops, they reported that they were often not provided with adequate information regarding the specific content, needs and interests of their medical learners, as well as their competencies and expectations. As one focus group participant clearly stated: “I really wasn’t given any information… outlines of what they needed [to know] … would have been helpful” (Dietitian, FG4).

Many felt that these essential components were missing when structuring their curricula. This lack of information of the medical learners’ current knowledge level was a recurring challenge for most, highlighting that – not having gone through medical school themselves – NPHCPs shared a fundamental need for information about their learners.

NPHCPs also reported being asked to provide education with minimal lead time, leading to the challenge of last-minute learner scheduling, inadequate preparation time for teaching and difficulty in scheduling appropriate patients.

NPHCPs found it challenging to give feedback to learners because they did not have formal roles as preceptors. As most educators did not have faculty status, they were lacking knowledge of department structure to voice concerns. There was no clear mechanism for NPHCPs to discuss difficulties with specific students. Furthermore, for teachers without faculty status, there was no system in place to receive formal feedback on their teaching. Participants unanimously desired feedback on their teaching and indicated they would use it for future educational planning. Often they relied on repeated requests for teaching sessions or changed learner behaviours as indirect positive feedback on their teaching contributions.

#### Preparation for an educator role

Focus group participants reported little to no training for teaching medical learners when they first took on their role as educators. Most subsequently participated in some educational opportunities to solidify or expand their skill sets including workshops, conferences and courses. NPHCPs reported that proficiency as educators increased with time and exposure. They also expressed a desire to participate in continuing education to further enhance their skills.

### II. Feeling valued as an educator

Participants reported that medical learners initially had a poor understanding of the NPHCP role as educator. It took time and exposure to NPHCPs for the learners to recognize their value and better appreciate their role NPHCP within their field and as educators in Family Medicine. “I’ve had to earn my stripes” (FG3, nurse).

NPHCP participants believed that their teaching contributions extended beyond clinical content to include providing education regarding appropriate team behaviors. The importance of teaching independent thinking and preparing learners for a practice that may or may not include interprofessional team members was recognized.

An ongoing challenge reported by participants was differentiating the role of expert in a field versus the role of collaborator. They perceived a lack of training regarding how to teach the role of collaborator, an essential component of all health care teams. This was especially challenging when trying to differentiate their role as a content expert.

Not all NPHCPs received financial compensation or protected time for their roles as educators and for related professional development (PD). This resulted in many feeling undervalued and unappreciated for their specialized skills and escalating demands on their time. Some participants reported that this affected their motivation both to continue teaching and to further seek PD opportunities.

### Faculty appointment

Of the 21 participants, 8 had faculty status through the DFCM. Focus group participants varied in their awareness of seeking faculty appointments as an option and of the benefits that such appointments could bring. Those without faculty status were very interested to learn about the benefits, including: PD opportunities, support for conference attendance, networking, library services, and access to resources. Intangible benefits, such as respect and legitimization of their role were also discussed as additional important aspects of faculty appointment.

The respondents who had faculty status and had taken advantage of associated PD opportunities in the past were grateful for these opportunities and felt that they contributed to their teaching roles. They also felt that this status had a positive effect on their acceptance and value placed on their teaching role by learners. However, many felt that most existing PD opportunities were geared towards physicians. Nevertheless, NPHCPs felt that those PD opportunities should be expanded to include all NPHCPs regardless of faculty status. The NPHCPs felt that having faculty status would also provide the opportunity for being mentored and solidifying their role in the teaching team.

### III. Competing demands

Participants viewed challenges relating to time, space and money as interlinked. Participants reported that having medical learners added additional time pressures. They felt internal conflict when taking on education responsibilities, as this directly affected patient care time. When time was limited, they often had to choose between taking medical learners or learners from their own professions, often with a preference for the latter. “If I can’t even give back to my own profession, how can I … educate other health care professionals …?” (FG 3, physiotherapist).

Participants also identified physical space as an important factor in improving their opportunity and quality of teaching. They felt that sharing space with residents would enhance involvement and increase interaction and appreciation of each other’s role. Unfortunately, ideal workspace was often unavailable. Limited space also affected their ability to appropriately teach and debrief learning experiences. In the busy and public workplace, there was sometimes no place to debrief privately with learners. Limited space was raised as a barrier that affected all types of NPHCP teaching, regardless of whether or not the learner was a medical learner.

## Discussion

The literature suggests that integrating NPHCPs into Family Medicine teaching may enhance medical trainees’ learning experience through exposure to rich interprofessional collaboration. As well, it may help in distributing medical education responsibilities, relieving some pressure from physicians already heavily involved in teaching. However, as our findings indicate, while NPHCPs may be actively involved in medical teaching, their roles are not necessarily appropriately planned, resourced or supported.

Better integration of NPHCPs into medical education is therefore needed to help ensure successful and sustainable cross-professional teaching practices. The limited literature on the role of NPHCP as medical educators proposes some potential solutions towards helping to ‘bridge the gap’ between current experience and optimal integration. For example, Clay et. al (2013) discuss the need for expending the traditional characterization of ‘faculty’ beyond just physician educators, and the importance of equipping all teachers in academic units with the language and skills to teach and learn in interprofessional settings [14]. Walsh et. al (2014) highlight a need for more intentional orientation of medical learners to the roles and scopes of NPHCP to help enable more effective clinical interactions and learning [8].

In this study, participants raised a number of similar strategies for enhancing integration of NPHCPs that have been informed by their own lived experience as educators in the largest Family Medicine program in Canada. First, our participants described a large degree of stress arising from receiving little or inadequate advance information on learner needs and the curricular context for teaching in both formal and informal education settings. For new NPHCP teachers, having more clearly defined learning objectives at the outset -- perhaps prepared individually by trainees and/or provided by a higher level in the institution -- would help the NPHCPs better target their teaching efforts to meet learner needs.

Second, many of the NPHCPs in this study discussed a desire to receive more feedback on, and formal evaluation of their teaching activities. Feedback on teaching performance has been shown to encourage educators in their work, acknowledging their successes and promote enhancement of their teaching skills. In this case, NPHCPs desired feedback in order to further refine their skills as teachers, clinical experts, and also collaborators. Simple revisions to current program and rotations evaluations would enable trainees to provide feedback on NPHCP teachers in addition to their usual physician preceptors.

Professional development and faculty status was another significant area of discussion amongst participants. Increasing the awareness of the value of faculty appointment as well as improving the ease with which NPHCPs may apply for status within an institution may be additional considerations for further supporting the NPHCP role in medical education. For example, securing faculty status may enhance the visibility of NPHCPs as medical educators at the level of the training institution, enable improved access to PD opportunities to enhance teaching, as well as engage NPHCPs in an establish community of practice of medical educators in primary care.

One of the barriers affecting the NPHCPs’ experience in teaching was their perceived value as medical educators. In this study, NPHCPs felt that while they were ultimately valued in their teaching, this did not occur until later in the teaching process. Once more aware of their professional roles, medical learners were more appreciative of the clinical expertise of NPHCP as well as appreciating the role NPHCPs played in collaboration and team provision of care. Teaching health care professionals interprofessionally is a primary educational strategy for training learners for practice in interprofessional collaborative health care settings [15]. We can extrapolate the contact hypothesis, where positive contact can improve poor student attitudes towards different groups [16,17].

NPHCPs in this study identified a number of resources that would support them in effectively implementing their roles as teachers, including protected teaching time to decrease the pressure from competing clinical demands, acknowledgement of their responsibilities to their own profession learners, and access to appropriate space. As with any medical educator, securing appropriate tangible and intangible resources such as having appropriate time for teaching preparation, financial remuneration and physical space for discussion is essential for optimal teaching activities. Creative strategies for securing these elements for NPHCP educators as available to their physician counterparts needs to be further considered to ensure the sustainability and effectiveness of their role as medical teachers.

Interestingly, findings from this study were seen by the authors as paralleling the progression of Family Medicine as an academic discipline within the Faculties of Medicine and universities over time. Thirty years ago, few family physicians had academic appointments. Medicine as a discipline has subsequently progressed to embrace Family Medicine teaching and research, with the value of this expanded role evident in the support and infrastructure that is now part of all Canadian Faculties of Medicine. Our findings in this study suggest that NPHCP educators within Family Medicine may be on a similar trajectory, as a sub-group facing historically similar barriers and desiring similar supports.

Anecdotally, the authors of this study were not collectively surprised by the findings. In their experience, the significant contribution of NPHCPs has been increasing steadily over the past decade in the University of Toronto DFCM program, as well as DFCMs across the country. Along with growing involvement in medical education, the desire for more support and the challenges encountered by NPHCPs in their teaching role have, to a certain extent been anticipated by departmental leadership. The tension NPHCPs reported experience when faced between teaching medical learners and teaching learners from their own profession with limited teaching time, however, was a new finding. It was also surprising to find the unanimous frustration in the lack of information provided about medical learner goals and expectations, as well as a lack of a formal method for giving feedback and evaluations. There has been minimal research demonstrating and articulating these challenges, and the findings highlight the need for more study [7].

This study has some limitations. Focus group participants were self-selected, in that only those interested and able to participate in the focus groups were involved. Participants who want to partake in a focus group are likely those with strong opinions or special interest. This may be reflected in the fact that that 38% of our participants had faculty status appointments, compared to only 18% respondents identified in the initial survey. Qualitative results are not intended to be generalizable. While this research was conducted on NPHCPs at a single training institution, this was partially mitigated as it involved participants from a wide range of health professional backgrounds working in diverse clinical settings.

## Conclusions

For NPHCPs to be involved in medical teaching in a successful and sustainable manner, they need to be appropriately supported, resourced and valued. This study provides guidance for enhancing the contribution of NPHCPs to medical education. As their involvement increases, it is essential to consider how best to support NPHCPs. Formal integration into their academic role, including regular education sessions, increased teacher-learner contact and discussion of learning needs and evaluation and faculty appointment may improve the education and collaborative experience of learners and educators. Ongoing needs assessment and continuing evaluation of NPHCP resources and support in medical teaching are essential to further guide this process. While this should be a standard process in all curriculums, NPHCPs’ less formal/integrated role as faculty may be contributing to the lack of what is widely accepted as required. NPHCPs value their roles as educators. The diverse learning experiences they provide will be beneficial to learners in their future careers as medical practitioners and collaborators.
